# Clinical and clinicopathological features and outcomes of cats with suspected dietary induced pancytopenia

**DOI:** 10.1111/jvim.16613

**Published:** 2023-01-07

**Authors:** Barbara Glanemann, Karen Humm, Mariana Abreu, Sophie Aspinall, David Buckeridge, Hope Carveth, Hannah Darcy, Jessica Florey, Polly Frowde, Isuru Gajanayake, Kate Green, Emma Holmes, Alenka Hrovat, Anne‐Katherine Jasensky, Bryn A Jones, Vasiliki Lantzaki, Eve JY Lo, Kirsty MacDonald, Kevin O'Brien, Alejandro Suárez‐Bonnet, Nele Van den Steen, Balazs Szladovits, Annelies Willems, Helen Wilson

**Affiliations:** ^1^ Department of Clinical Science and Services Royal Veterinary College Hatfield UK; ^2^ Medivet 24hour Alder Liverpool UK; ^3^ Paragon Veterinary Referrals Wakefield UK; ^4^ Synlab VPG Exeter UK; ^5^ CheshirePet Veterinary Practice UK; ^6^ London Veterinary Specialists London UK; ^7^ Dick White Referrals Six Mile Bottom UK; ^8^ Davies Veterinary Specialists Hitchin UK; ^9^ Willows Veterinary Centre & Referral Service Solihull UK; ^10^ Moorview Referrals UK; ^11^ Department of Pathobiology and Population Sciences Royal Veterinary College Hatfield UK; ^12^ Pride Veterinary Centre Derby UK; ^13^ Anderson Moores Veterinary Specialists Winchester UK; ^14^ School of Veterinary Medicine Small Animal Hospital, College of Medical, Veterinary, and Life Sciences, University of Glasgow Glasgow UK; ^15^ Drove Veterinary Hospital Swindon UK; ^16^ Wilson and Partners, Cupar Fife UK; ^17^ Cave Veterinary Specialists Wellington UK; ^18^ The Ralph Veterinary Referral Centre Marlow UK; ^19^ Langford Vets University of Bristol Bristol UK

**Keywords:** aplastic anemia, neutropenia, T‐2/HT‐2 mycotoxin, thrombocytopenia, trichothecenes

## Abstract

**Background:**

After a strong epidemiological link to diet was established in an outbreak of pancytopenia in cats in spring 2021 in the United Kingdom, 3 dry diets were recalled. Concentrations of the hemato‐ and myelotoxic mycotoxins T‐2, HT‐2 and diacetoxyscirpenol (DAS) greater than the European Commission guidance for dry cat foods were detected in the recalled diets.

**Objectives:**

To describe clinical and clinicopathological findings in cats diagnosed with suspected diet induced pancytopenia.

**Animals:**

Fifty cats presenting with pancytopenia after exposure to a recalled diet.

**Methods:**

Multicenter retrospective case series study. Cats with known exposure to 1 of the recalled diets were included if presented with bi‐ or pancytopenia and underwent bone marrow examination.

**Results:**

Case fatality rate was 78%. Bone marrow aspirates and biopsy examination results were available in 23 cats; 19 cats had a bone marrow aspirate, and 8 cats had a biopsy core, available for examination. Bone marrow hypo to aplasia—often affecting all cell lines—was the main feature in all 31 available core specimens. A disproportionately pronounced effect on myeloid and megakaryocytic cells was observed in 19 cats. Myelofibrosis or bone marrow necrosis was not a feature.

**Conclusion and Clinical Importance:**

Mycotoxin induced pancytopenia should be considered as differential diagnosis in otherwise healthy cats presenting with bi‐ or pancytopenia and bone marrow hypo‐ to aplasia.

**Abbreviations**
DAS: diacetoxyscirpenol
FELV: feline leukemia virus
FIV: feline immunodeficiency virus
FPLV: feline panleukopenia virus
FSA: Food Standard Agency
LOAEL: lowest observed adverse effect level
NOAEL: no observed adverse effect level

## 1 INTRODUCTION

In spring 2021 an outbreak of pancytopenia in cats occurred in the United Kingdom and investigations into an underlying cause revealed a strong epidemiological link to diet leading to the recall of 3 commercial feline diets.^1^ Analysis of representative feed samples from recalled diet brands revealed a concentration of the trichothecene mycotoxins T‐2 and HT‐2 up to 5 times higher than recommended by the European Commission.^2^

Trichothecene mycotoxins (including T‐2, HT‐2, and diacetoxyscirpenol) belong to the largest group of Fusarium mycotoxins, and at present more than 170 different trichothecenes have been isolated.^3^, ^4^ The hemato‐ and myelotoxicity of trichothecenes, in particular of T‐2 and HT‐2 toxin but also of DAS, is well recognized in the literature, mainly attributed to their inhibitory effect on protein, RNA and DNA synthesis, induction of ribotoxic stress and apoptosis, resulting in impaired hematopoiesis in the bone marrow.^3^, ^4^, ^5^ T‐2 and HT‐2 are generally associated with grain products and are most frequently and at their highest concentration found in oats and oat products.^4^ Ingestion of grains contaminated with T‐2 and HT‐2 toxin are the underlying cause for human alimentary toxic aleukia, a disease characterized by severe bone marrow hypocellularity and high mortality rate.^6^, ^7^, ^8^

Experimental studies in cats demonstrate the potential toxic, dose‐dependent fatal effect of T‐2 toxin on cats. Clinical signs before death include lethargy, anorexia, hemorrhagic diarrhea and weight loss, and postmortem examination reveals multifocal hemorrhages in several organs with markedly decreased cellularity of the bone marrow and resembling the disease in humans.^9^, ^10^, ^11^ Despite these studies, detailed toxicological data allowing the establishment of a specific *lowest observed adverse effect level* (LOAEL) or *no observed adverse effect level* (NOAEL) for cats are lacking.^5^, ^12^

The aim of this study was to describe the clinical and clinicopathological findings of cats with suspected dietary derived mycotoxin induced pancytopenia presented during the outbreak in 2021.

## 2 MATERIAL AND METHODS

This is a retrospective, multicenter, case series study; ethical approval was granted by the Royal Veterinary College, University of London ethics and welfare committee (URN SR2021‐0148).

### 2.1 Study cohort

Cats were considered to be eligible for inclusion if they presented with a neutropenia (neutrophil concentration <2.5 × 10^9^/L) or thrombocytopenia (<150 × 10^9^/L), or both with or without anemia (hematocrit or packed cell volume <27%) between February 1st and December 11th 2021. Additionally, cats had to have had exposure to 1 of the recalled diets as well as undergone microscopic bone marrow examination of diagnostic quality.

Data collected included date of presentation, signalment, duration and type of clinical signs, indoor/outdoor status, laboratory and body cavity imaging findings, bone marrow examination results, feline immunodeficiency, feline leukemia and feline panleukopenia virus testing results, other diagnostics and dietary information for each cat affected.

### 2.2 Statistical analysis

Analyses were conducted using SPSS version 28.0 (IBM Corp). If continuous variables were non‐normally distributed, they were summarized using median and range. Normally distributed, continuous variables were presented as mean and SD.

## 3 RESULTS

### 3.1 Demography

Fifty cats presenting to 14 veterinary referral and 3 veterinary university hospitals were eligible for inclusion. The cats were presented between March 5th to July 18th 2021. The median age at first presentation was 17 months (range, 3‐159). Breeds included domestic shorthair (29; 58%), British shorthair (5; 10%), ragdoll (5; 10%), domestic longhair (3; 6%), 2 Maine coon cats and crossbreeds, and 1 each of the following: Abyssinan, domestic medium hair, sphynx and Oriental shorthair. Of the cats, 20 (40%) were neutered males, 20 (40%) neutered females, 5 (10%) entire males, and 5 (10%) entire females.

### 3.2 Cat characteristics, clinical signs, and initial diagnostics

There were 26 (52%) cats that were housed exclusively indoors, and 23 (46%) that had indoor and outdoor access. Indoor/outdoor status was unknown for 1 cat. There were 19 (38%) cats from a single cat household, and 31 (62%) were from a multicat household. Of the cats from a multicat household, other cats in the household were diagnosed with bi‐ or pancytopenia in 19 (61%) cases. Thirteen cats (26%) were fed diet A, 23 (46%) cats diet B, and 14 cats (28%) diet C.^1^ Twenty‐five cats (50%) were recorded as being fed solely on a dry diet, whereas 22 cats (44%) were fed a combination of wet and dry.

The median duration of clinical signs before presentation was 2.0 days (range, 0‐61). The most common clinical signs reported were lethargy (34; 68%), inappetence (28; 56%), petechiae (18; 36%), pyrexia (16; 32%), pale mucous membranes (13; 26%), and hematochezia (11; 22%).

The median total white blood cell concentration was 1.10 × 10^9^/L (range, 0.27‐24.05), median neutrophil concentration 0.03 × 10^9^/L (range, 0.00‐21.60), median platelet concentration (confirmed by slide examination) 3.0 × 10^9^/L (range, 0.0‐77.0) and median PCV 17.0% (range, 5.0‐35.0). The median lymphocyte concentration was 0.96 × 10^9^/L (range, 0.23‐5.87) and median eosinophil concentration was 0.03 × 10^9^/L (range, 0.00‐0.80).

Serum biochemical data were available for analysis in 46 cats. Median bilirubin concentration was 5.0 μmol/L (range, 1.0‐49.0). Other measurements are presented in Table 1.

**TABLE 1:** Biochemical data from 46 cats with pancytopenia

Variable	Result	Reference range
Albumin (g/dL)	2.44 ± 0.38	2.5‐4.5
Globulin (g/dL)	3.47 ± 0.69	2.5‐4.5
Urea (mg/dL)	44.4 (20.4‐312)	15.0‐59.5
Creatinine (mg/dL)	0.9 ± 0.28	0.2‐2.0
Bilirubin (mg/dL) (n = 44)	0.29 (0.06‐2.92)	0.01‐0.3
ALT activity (U/L)	34.6 (11‐171.20)	5.0‐60.0
ALP activity (U/L)	10.0 (0.0‐108.0)	0‐60.0

The result of feline immunodeficiency virus antibody and leukemia virus antigen (FIV, FeLV; SNAP FIV/FeLV Combo Test, IDEXX) testing was negative in all tested cats (42/50). Eight cats had FeLV proviral DNA by polymerase chain reaction (PCR) testing performed on bone marrow aspirate samples; all were negative. Feline panleukopenia virus (FPLV) testing on blood and fecal samples was negative in all tested cats (8/50 and 9/50, respectively). PCR testing for FPLV was performed on 14 bone marrow aspirates; 12 tested negative and 2 weakly positive (cycle threshold [Ct] >31).

Diagnostic imaging of body cavities was performed in 23 (46%) cats with no clinically important abnormalities detected. Imaging was not performed in 28 (56%) cats of which 17 (61%) cats underwent postmortem examination.

Postmortem examination of the 17 cats revealed mainly diffuse, multifocal hemorrhages in several organs as well as evidence of sepsis in a few cases. No etiological cause for the severe pancytopenia was identified.

### 3.3 Bone marrow findings

A bone marrow aspirate and core sample of adequate diagnostic quality were available for microscopic evaluation in 23 cats, 19 cats had an aspirate sample only, and 8 cats had a core sample only available. Bone marrow specimens were obtained at time of euthanasia or as part of a postmortem examination in 18 (36%) cats; all other cats underwent bone marrow aspiration and biopsy as part of their diagnostic work‐up.

**FIGURE 1:**
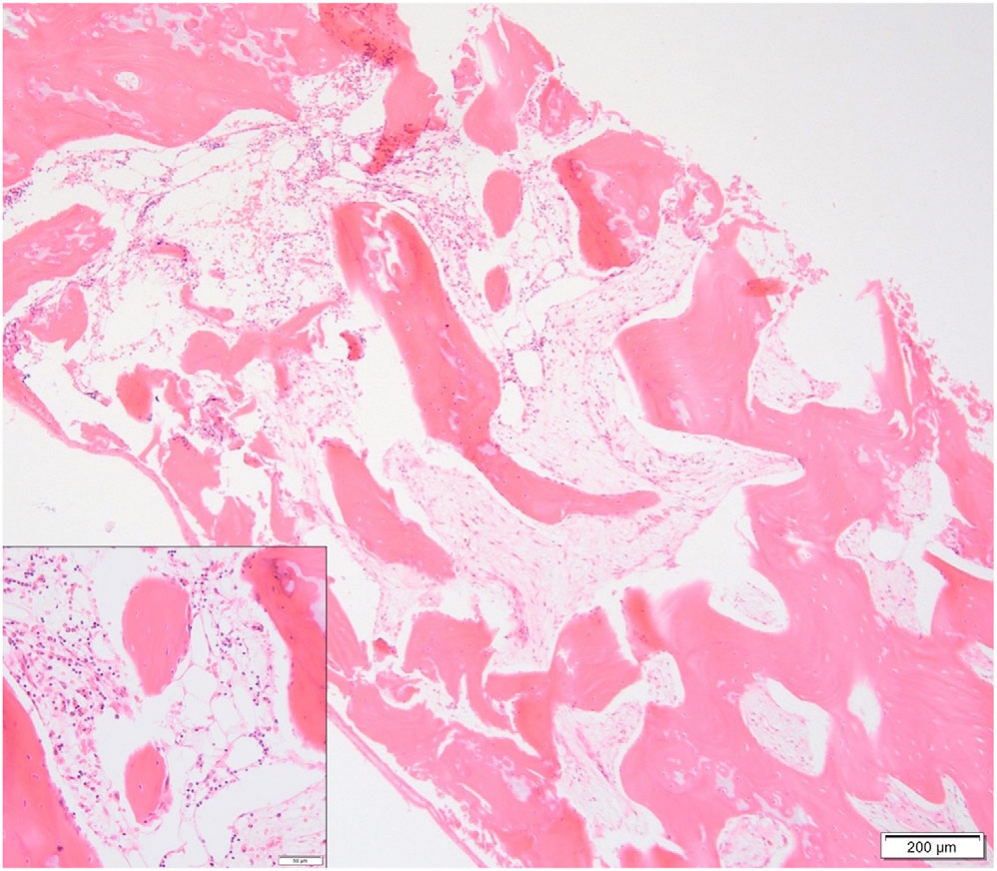
Core section of feline bone marrow with marked generalized hypoplasia. H&E stain, 4× objective, bar 200 μm. Insert: Close up from an area with low numbers of remaining segmented cells (eosinophils mostly). H&E stain, 20× objective, bar 50 μm

Of the cats with aspirate and core samples available for examination, biopsy results were consistent with aplastic anemia in 14 (61%) cats; 8 (35%) cats had moderately to severely hypocellular bone marrows (Figure 1) and 1 (4%) cat was suspected to have a mildly hypercellular bone marrow due to erythroid hyperplasia with concurrent marked myeloid and megakaryocytic hypoplasia. In 14 (61%) of 23 cats, aspirate examinations were suggestive of a disproportionate effect on cells of the myeloid and megakaryocytic lineage compared to cells of the erythroid lineage; 3 cats had erythroid hyperplasia and 2 cats had erythroid normoplasia with concurrent marked hypo to aplasia of cells of myeloid and megakaryocytic lineage.

**FIGURE 2:**
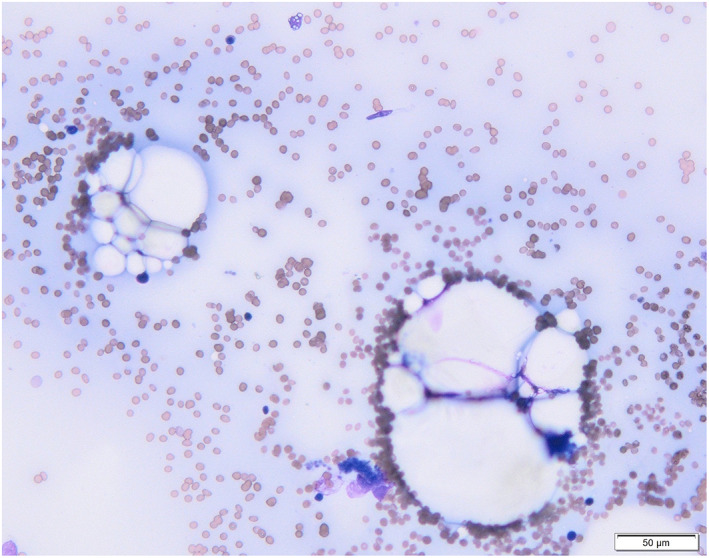
Smear form an aspirate of feline bone marrow with marked generalized hypoplasia. Spicules contain mostly adipocytes. Wright's stain, H&E stain, 20× objective, bar 50 μm

Of the cats with only an aspirate available for examination (19), all but 1 were diagnosed with either suspected generalized bone marrow aplasia or hypoplasia (Figure 2). Two of these cats had a disproportionate effect on cells of myeloid and megakaryocytic lineage with myeloid and megakaryocytic aplasia, erythroid hypoplasia and concurrent left shift. A further 2 cats were reported with erythroid and myeloid hypoplasia with concurrent megakaryocytic aplasia. One cat had megakaryocytic and erythroid hypoplasia but myeloid hyperplasia.

Five of the 8 cats with only bone marrow core samples were diagnosed with severe hypo‐ to aplasia (63%) affecting all 3 cell lineages; 1 cat was diagnosed with megakaryocytic aplasia, myeloid hypoplasia and mild erythroid hyperplasia and 2 cats with erythroid and myeloid hypoplasia with concurrent megakaryocytic aplasia. Reports of bone marrow cores of 3 cats specified aplastic anemia.

Evidence of myelofibrosis was suspected in 1 cat (2%) and in the same cat large areas of granular streaming amorphous necrotic marrow tissue was present and individual cell necrosis was evident on bone marrow core examination. No evidence of necrosis or myelofibrosis was detected in any of the other samples.

The presence of lymphocytes (small or mixed populations) within the bone marrow was specifically mentioned in 38 bone marrow specimens, all of which had generalized bone marrow hypo‐ or aplasia. Other space occupying processes were not reported in any cases. In 18 bone marrow specimens with marked myeloid hypoplasia, the scant myeloid precursors were mainly of eosinophilic lineage. Eosinophilic normoplasia was suspected in 1 cat.

Features of erythroid dysplasia were described in 15 bone marrow aspirate specimens, 3 of those with concurrent erythroid normoplasia to mild erythroid hyperplasia, all others with concurrent moderate to severe erythroid hypoplasia.

### 3.4 Treatment and outcome

Thirty‐nine (78%) cats did not survive to discharge (hospitalization time median 2 days; range, 0‐17 days). Hospitalization time for the 11 surviving cats ranged from 1 to 26 days (median 12 days). In total, 35 (70%) cats received blood products during the initial presentation. Of the cats that survived for at least 24 hours after presentation (40 cats; 80%), 32 (80%) received blood products. Xenotransfusion was performed in 18 cats, with 13 of those also either receiving a feline packed red blood cell (pRBC) unit (9/18), a feline whole blood transfusion (2/18) or feline fresh frozen plasma (2/18). In total 26/35 cats received a feline pRBC unit, with a median of 1 unit/cat (range, 1‐3). Of the 18 cats receiving xenotransfusion, 3 cats survived to discharge. Of the 17 cats receiving feline pRBC, whole blood, or both, 3 cats survived to discharge. Other treatment administered included broad‐spectrum antibiotics (46 cats; 92%), glucocorticoids (29 cats; 58%), filgrastim (11 cats; 22%), tranexamic acid (12 cats; 24%), ciclosporine (4 cats; 8.0%), cobalamin supplementation (10 cats; 20%), gastroprotectant and antiemetic medication (7 cats each; 14%), opioids for pain management (6 cats; 12%) and vincristine, vitamin K and vitamin E supplementation (1 cat each). Median prednisolone equivalent dose of administered glucocorticoid was 1.5 mg/kg BW/day (range, 0.5‐4.0).

## 4 DISCUSSION

This study describes clinicopathological features, in particular bone marrow examination findings, of cats presenting with pancytopenia that were exposed to a diet containing the trichothecene mycotoxins T‐2, HT‐2, and diacetoxyscirpenol (DAS) in higher concentrations than advised.^2^ Demographics, duration and type of clinical signs, results of hematological and biochemical analysis, as well as infectious disease testing results of presented cats in this study did not differ from the cohort involved in the epidemiological investigation of the outbreak of pancytopenia in cats in the United Kingdom,^1^ and therefore this case series can be presumed to be a representative sample.

Assessment of bone marrow cores was suggestive of aplastic pancytopenia (formerly also called aplastic anemia) in 55% of cats (17 of 31 cats with a bone marrow core sample being available). The remaining bone marrow specimens were generally characterized by marked hypocellularity with an increased amount of fat, apart from 1 specimen that was classified as mildly hypercellular (60% nucleated cells in marrow spaces; 5 year old FN DSH) due to marked erythroid hyperplasia with concurrent marked myeloid and megakaryocytic hypo‐ to aplasia. Aplastic pancytopenia, characterized by multiple, often severe, cytopenias in the peripheral blood, with concurrent generalized bone marrow hypo‐ to aplasia and replacement of hematopoietic precursor cells by adipose tissue, is a relatively rare condition in cats.^13^ Described underlying causes including *FeLV*, *FIV* infection and exposure to myelotoxic drugs; including chemotherapeutics, griseofulvin, trimethroprim/sulfadiazine, methimazole were excluded.^14^ *Ehrlichia* sp. infection as an underlying cause was ruled out in only 1 of the cats, though as this infectious agent is not endemic in the United Kingdom it is considered an unlikely cause.^14^ PCR performed on bone marrow aspirate was positive for panleukopenia virus in 2 cats; though both positive results were only obtained after a high cycle threshold suggesting a low viral load being present. Additionally, the clinical presentation of cats was not suggestive of feline panleukopenia virus infection.^15^ Estrogen toxicosis was ruled out in only 1 cat but was not suspected clinically due to a lack of compatible history in any of the cats.^16^

Hemato‐ and myelotoxic effects of T‐2 and HT‐2 have been described in various species.^3^ T‐2 toxin affects human hematopoietic progenitor cells rather than mature, circulating blood cells and the toxic effects are attributed to inhibition of hematopoiesis.^17^ Inhibition of hematopoiesis is mediated by apoptosis of CD34+ stem cells.^18^ Red blood cell progenitors appear less sensitive to the toxic effect of T‐2/HT‐2 compared to platelet and white blood cell progenitors.^12^, ^19^

A disproportionate effect on cells of myeloid and megakaryocytic lineage, often with a left shift in the erythroid lineage, was noted in our cohort, a finding that is not typical for aplastic pancytopenia; this might be suggestive of feline erythroid precursors also being less sensitive to the toxic effect of T‐2 and HT‐2 toxin, compared to the myeloid and megakaryocytic lineage. This finding could also potentially represent different time points of sampling regarding duration and severity of toxin exposure for the different cats in the study. We suspect that the apparent selective effect on cells of myeloid and megakaryocytic lineage reflects an earlier stage of the disease process rather than the targeting of cells of 1 or 2 different lineages specifically. This could be further supported by the fact that in several of those cases blast stages were rarely identified across all 3 cell lineages, even in cases of erythroid hyperplasia. Due to a lack of detailed dietary information on amount and duration of ingestion of affected feed types, dose‐dependent effects are also possible, but could not be further investigated in our cohort.

The dysplastic changes of the erythroid precursors observed in 15 cats were considered to reflect secondary dysmyelopoiesis, likely as a consequence of the disruptive process in the bone marrow and accelerated production of the erythroid precursors, rather than primary myelodysplasia. Dysplasia of cells of other lineages were not reported.

One cat interestingly presented with severe thrombocytopenia and anemia but mild to moderate neutrophilia. Bone marrow examination in this cat revealed marked megakaryocytic and erythroid hypoplasia with concurrent myeloid hyperplasia—a feature that had not been seen in any of the other cats. Earlier reports of human alimentary toxic aleukia described 4 stages of disease, where in the first stage the total number of peripheral leukocytes either does not change or increases slightly and a similar effect is seen in pigs administered DAS toxin.^20^, ^21^ Alternatively, this cat might have had nonassociative immune hemolytic anemia (IMHA) and immune thrombocytopenia (ITP). Evan's syndrome is a rare condition in cats and no data on bone marrow findings for cats with this syndrome is available.^22^ Erythroid and megakaryocytic hyperplasia might be expected in these cases, though amegakaryocytic thrombocytopenia has been described in dogs with ITP.^23^ The cat did not show any evidence of hemolysis (eg, ghost cells, hyperbilirubinemia) and peripheral evidence of immune destruction of red blood cells were not reported or performed (eg, In Saline Agglutination, Coombs’ testing), although immune destruction of erythroid precursors could result in erythroid hypoplasia.

In several cats the myeloid lineage appeared to have selective disruption, specifically of the neutrophil and monocyte lineage. If myeloid precursors were present, the predominating cell type was of eosinophilic lineage. It is not possible to rule out whether those eosinophils were prominent due to a specific cytokine driven mechanism, or present in normal numbers and just not affected similarly as the other cell lineages. The presence of eosinophils on bone marrow examination is described in pigs being administered DAS but not on bone marrow specimens from dogs in the same study.^21^ Pigs and dogs, both developed severe cellular necrosis in the hematopoietic cords when given DAS, and severity of necrosis appeared to be dose‐related.^21^ Necrosis was reported in 1 of the 50 cats in our study.

T‐2 toxin is able to induce hemolysis in some animal species (eg, rabbits, dogs, horses, and humans) with variable degree of effect, whereas ruminants seem to be resistant to this effect due to degradation of T‐2 toxin and related toxins by rumen microbiota.^12^ Evidence of hemolysis based on mismatching hemoglobin concentration and PCV or hyperbilirubinemia was not a feature in the presented group of cats. Eight cats (16%) had mild to moderate increases in serum bilirubin concentration, though this was considered most likely due to functional cholestasis associated with severe inflammatory disease/sepsis.^24^

Diacetoxyscirpenol has a similar myelo‐ and hematotoxic effect compared to T‐2/HT‐2 and in pigs it is more toxic than T‐2.^21^, ^25^ Like T‐2/HT‐2 it is extensively absorbed from the gastrointestinal tract and rapidly excreted via urine and feces within 12 to 24 hours.^26^ The extensive metabolism of DAS is mediated by four pathways—similar to T‐2/HT‐2—by means of hydrolyzation, hydroxylation, de‐epoxidation and conjugation, with the latter pathway lacking in felids.

Cats are considered to be a sensitive species in regard to the toxic effects of T‐2/HT‐2 toxin which is likely associated with their inability to excrete T‐2 toxin and its metabolites via glucuronide conjugation.^5^ Therefore, it has to be assumed that a similar sensitivity exists toward the DAS toxin.

Case fatality rate in this study was higher compared to the case fatality rate reported for the overall affected cohort.^1^ This is thought to be due to 2 main factors. One, the reported case fatality rate for the overall affected cohort is likely an underestimation due to the nature of reporting and capture of data (“1 moment only”) in that study and also due to the fact that it is very likely that not all affected cats were registered on our data base.^1^ In contrast, the case fatality rate reported here is likely an overestimation, as availability of bone marrow examinations results was an inclusion criterion. Bone marrow aspiration and biopsy as part of the diagnostic work‐up were mainly performed on cats presenting before the food was recalled, whereas the majority of bone marrow examinations performed after the food was recalled were performed only in association with euthanasia. After a strong link to diet had been established, cats presenting with pancytopenia and known exposure to 1 of the recalled diets were only minimally investigated and only underwent supportive treatment at the Queen Mother Hospital for Animals, Royal Veterinary College, for example, 11 of 13 cats underwent bone marrow investigations as part of their diagnostic work‐up, whereas only 9 of 26 cats presenting later only underwent bone marrow aspiration/core biopsy at time of euthanasia, with 65% of the cats presenting later surviving to discharge.

A limitation of this descriptive report is that obtaining a bone marrow sample of good diagnostic quality is more challenging in cats compared to some other species such as dogs and diagnosing hypocellular bone marrows is limited when analyzing solely aspirate samples. As reports were generated by numerous pathologists, a degree of heterogeneity was present in the way individuals classified findings as hypo or aplastic and not all reports mentioned certain key features.

Due to the lack of NOAEL and LOAEL for trichothecene toxins in cats, it remains speculative whether the pancytopenia in presented cats was caused by the mycotoxins T‐2/HT‐2 and DAS. However, similarities in clinical presentation and clinicopathological findings, in particular bone marrow examination findings compared to other species and reported studies in cats,^9^, ^10^, ^11^ as well as exclusion of other previously documented causes, make a causative link likely and veterinarians should consider dietary derived mycotoxin induced marrow toxicosis as a possible underlying cause in cats presenting with pancytopenia. It is interesting to note that all affected brands were marketed as grain‐free, which could potentially lessen the likelihood of mycotoxin contamination on initial consideration. Occurrence of trichothecenes mycotoxins is also described in vegetable, including starchy roots vegetable like potatoes.^2^, ^27^, ^28^ The diets associated with the pancytopenia outbreak in cats in the United Kingdom all contained potatoes flakes which, in this scenario, are thought to have been the likely source of mycotoxin.

## CONFLICT OF INTEREST DECLARATION

Authors declare no conflict of interest.

## OFF‐LABEL ANTIMICROBIAL DECLARATION

Authors declare no off‐label use of antimicrobials.

## INSTITUTIONAL ANIMAL CARE AND USE COMMITTEE (IACUC) OR OTHER APPROVAL DECLARATION

Approved by the Royal Veterinary College ethics and welfare committee (URN SR2021‐0148).

## HUMAN ETHICS APPROVAL DECLARATION

Authors declare human ethics approval was not needed for this study.
